# Prévalence et facteurs prédicteurs des troubles post-traumatiques chez les accidentés de la voie publique

**DOI:** 10.11604/pamj.2024.47.89.38015

**Published:** 2024-02-26

**Authors:** Rim Feki, Lobna Zouari, Yosra Majdoub, Sana Omri, Imen Gassara, Najeh Smaoui, Manel Maalej Bouali, Jihene Ben Thabet, Nada Charfi, Mohamed Maalej

**Affiliations:** 1 Service de Psychiatrie C, Centre Hospitalier Universitaire Hédi Chaker Sfax, Faculté de Médecine de Sfax, Sfax, Tunisie,; 2 Service de Médecine Préventive et Sociale, Faculté de Médecine de Sfax, Sfax, Tunisie

**Keywords:** Accident de la voie publique, état de stress aigu, trouble stress post-traumatique, dépression, road accident, acute stress disorder, post-traumatic stress disorder, coping, depression

## Abstract

**Introduction:**

les troubles post-traumatiques survenant à la suite d'un accident de la route ont un impact tant sanitaire qu'économique.

**Méthodes:**

notre étude prospective, vise à déterminer la prévalence de ces troubles, et à dégager leurs facteurs de risque auprès de sujets victimes d'accidents de la route et hospitalisés au service de chirurgie orthopédique et de traumatologie du Centre Hospitalo-Universitaire de Sfax-Tunisie.

**Résultats:**

soixante-dix sujets ont été inclus dans notre étude. La prévalence de l'état de stress aigu était de 37,1% et il a été associé au sexe féminin, au niveau scolaire bas, à la présence d'antécédents médicochirurgicaux, à la passivité au cours de l'accident, à la sévérité des lésions, et à la présence d'une symptomatologie anxieuse et dépressive. Le trouble stress post-traumatique était constaté chez 40% des sujets et il a été associé au milieu de résidence urbain, à la passivité au cours de l'accident, et à la symptomatologie anxieuse et dépressive. Des scores faibles des stratégies de coping fonctionnelles et des scores élevés des stratégies de coping dysfonctionnelles ont été significativement associés à ces deux troubles. Le niveau scolaire bas, la résidence en milieu urbain, un niveau élevé d'anxiété et de dépression et la stratégie de coping de déni apparaissent comme des facteurs de risque indépendants de l'état de stress aigu et du trouble stress post-traumatique.

**Conclusion:**

il s'avère ainsi important de déterminer un profil de personnes plus exposées aux troubles post-traumatique afin de permettre un dépistage précoce par les médecins avec lesquels les accidentés pourraient avoir des contacts dans les suites de leurs accidents.

## Abstract (en)

**Introduction:**

trauma-related disorders following a road accident have both a health and an economic impact.

**Methods:**

we conducted a prospective study to determine the prevalence of these disorders, and to identify risk factors in subjects victims of road accidents and hospitalized in the Department of Orthopedic Surgery and Traumatology of the University Hospital Center of Sfax-Tunisia.

**Results:**

a total of sixty-ten subjects were included in this study. The prevalence of acute stress disorder was 37.1% and was associated with female sex, low educational level, previous medical and surgical history, passivity during the accident, severity of injuries and the presence of anxious and depressive symptoms. Post-traumatic stress disorder was observed in 40% of subjects and was associated with urban residential environment, passivity during the accident and anxious and depressive symptoms. Low scores for functional coping strategies and high scores for dysfunctional coping strategies were significantly associated with both disorders. Low educational level, urban residential environment, high levels of anxiety and depression, and denial coping strategy appear to be independent risk factors for acute stress and post-traumatic stress disorder.

**Conclusion:**

It is therefore important to determine the profile of people at greater risk of post-traumatic stress disorder, to enable early diagnosis in victims of road accidents.

## Introduction

Le fléau des accidents de la circulation (AC) constitue un enjeu majeur de santé publique et pose un problème social et économique. Ces accidents causent des séquelles physiques avec une détérioration des conditions de vie. Selon l'Organisation mondiale de la Santé, les AC ont entrainé 20 à 50 millions de blessés dans le monde durant l'année 2020 [1]. La Tunisie est classée la vingtième en Afrique en termes de mortalité routière avec un taux de 24,11% pour 100 000 habitants en 2015 [1]. D'après les chiffres communiqués par l'Observatoire National de la Sécurité Routière (ONSR), 4726 accidents ont eu lieu durant l'année 2020, ayant causé 938 décès et 6719 blessés. L'impact traumatique de l'AC; par son aspect inattendu et involontaire; est depuis longtemps reconnu. Ses conséquences psychologiques les plus fréquentes sont l'anxiété, la dépression, le trouble stress aigu (TSA) ou l'état de stress aigu (ESA) du DSM-IV et le trouble stress post-traumatique (TSPT) [2]. Dans le DSM 5, le TSA concerne une période transitoire de trois jours à un mois après le traumatisme et associe des symptômes appartenant aux cinq catégories suivantes: symptômes envahissants, humeur négative, symptômes dissociatifs, symptômes d'évitement, et des symptômes d'éveil. L'introduction du TSA a permis de prédire l'évolution vers un TSPT [3]. Les symptômes du TSPT doivent survenir un mois après l'événement et entrainer un dysfonctionnement dans la vie ou un niveau pathologique de détresse. Ils incluent la réviviscence de l'événement traumatique, l'évitement des éléments qui rappellent cet événement, une altération négative des cognitions et de l'humeur et une altération marquée de l'éveil et de la réactivité débutant ou s'aggravant après la survenue de l'événement traumatique [4]. Plusieurs facteurs favorisant la survenue de troubles post traumatiques ont été identifiés. Les stratégies de coping semblent avoir un impact non négligeable dans le développement de ces troubles mais elles restent peu abordées dans la littérature [5]. Dans ce travail, nous nous sommes donnés pour objectifs de déterminer la prévalence de l'ESA et du TSPT dans une population d'accidentés de la circulation, de dégager les facteurs de risque de survenue de ces troubles et d'étudier les stratégies de coping utilisées pour faire face au traumatisme psychique.

## Méthodes

**Cadre et type de l'étude**: une étude prospective, descriptive et analytique, réalisée en deux temps (nommés respectivement T1 et T2), a été menée durant la période allant du 1^er^ septembre au 30 novembre 2020. Notre étude s'est déroulée au service de chirurgie orthopédique et traumatologie et à la consultation externe correspondante du Centre Hospitalo-Universitaire de Sfax, Tunisie.

**Participants de l'étude**: nous avons inclus dans notre série les sujets âgés de plus de 18 ans, hospitalisés à la suite d'un AC qu'elle soit la gravité du traumatisme, et après un délai minimum de 03 jours par rapport à l'accident. Les critères de non-inclusion ont été le refus de participer à l'enquête, les sujets ayant une déficience mentale, et les traumatisés crâniens sévères (du fait que les lésions cérébrales peuvent donner des troubles psychiques d'origine organique). L'investigateur s'est déplacé au service concerné et a assuré la collecte des données à l'aide d'une fiche sous format papier incluant les différentes données sociodémographiques et cliniques ainsi que les échelles psychométriques.

**Conception de l'étude et instruments utilisés**: la première évaluation (T1) s'est déroulée après un recul minimal de 3 jours et au cours de la première semaine par rapport à l'accident. Au cours de cette évaluation, nous avons administré un questionnaire recueillant les données sociodémographiques, les antécédents personnels, et les données relatives à l'accident. L'échelle d'évaluation de la gravité du traumatisme AIS *(Abbreviated Injury Scale)* a permis de coder les lésions initiales de chaque victime grâce à un score de gravité qui répartit les lésions en mineures, modérées, sérieuses, sévères, critiques et maximales. Nous avons établi le bilan lésionnel à partir des données de l'examen clinique et des explorations paracliniques inclus dans les dossiers médicaux. Cette échelle a été développée pour fournir aux chercheurs une méthode numérique simple pour hiérarchiser et comparer les blessures par degré de sévérité ainsi que pour standardiser la terminologie décrivant les lésions. Les lésions sont considérées comme graves si le score AIS est supérieur ou égal à 3 [6]. L'échelle d'évaluation de l'état de stress aigu *(The Acute Stress Disorder Scale: ASDS)* est un auto-questionnaire comprenant 19 items basés sur les critères diagnostiques du DSM-IV de l'état de stress aigu et répartis en 4 sous-échelles: dissociation, reviviscence, évitement et hyperactivité neurovégétative. Le score seuil utilisé est de 56 [7]. La passation a été réalisée en langue française avec des explications en arabe des items incompréhensibles (une traduction à la langue arabe a été faite au préalable). L'échelle Brief COPE *(The Coping Orientations to Problems Experienced)* est composée de 28 items répartis en 14 sous-échelles évaluant les dimensions du coping. Ce test évalue les réponses au stress sur une échelle en 4 points (de ‘pas du tout' à ‘tout à fait') allant de réponses adaptées à des réponses dysfonctionnelles. Des scores plus élevés sur une sous-échelle indiquent une utilisation accrue de ce mécanisme d'adaptation. Muller *et al*. proposent une distinction entre stratégies fonctionnelles (coping actif, planification, acceptation...), et stratégies dyfonctionnelles (blâme, consommation de substances...) [8]. Il a été administré en langue française avec des explications des items non compris par les sujets interrogés. Tous les items ont été traduits au préalable en langue arabe. La deuxième évaluation (T2) a eu lieu 4 à 6 semaines après T1 au cours de la consultation de contrôle selon les aléas des rendez-vous. L'investigateur a administré l'échelle *HADS (Hospital Anxiety and Depression scale)* dans sa version validée en langue arabe par Terkawi, *et al*. en 2017 [9]. Elle est composée de sept items se rapportant à l'anxiété et sept autres à la dimension dépressive. Une symptomatologie anxieuse ou dépressive certaine a été retenue si le score est supérieur ou égal à 11 [9]. L'échelle *PCL-5 (Post-Traumatic Stress Disorder Checklist Scale)* est un auto-questionnaire en langue française de 20 items qui évalue les symptômes du TSPT selon les critères du DSM-5. Une valeur seuil de 38 suggère la présence d'un TSPT [10].

**Analyse des données**: nous avons utilisé le logiciel *SPSS (Statistical Package for the Social Sciences)* dans sa vingtième version. La comparaison entre les sous-groupes (ESA (+) et ESA (-); TSPT (+) et TSPT(-)) a été effectuée en utilisant le test de Chi2, le test t de Student pour les variables qualitatives (sexe, niveau scolaire, etc…), et le test de Mann-Whitney pour les variables quantitatives de distribution non gaussienne (âge, stratégies de coping). Enfin, une analyse de régression logistique en utilisant la méthode «pas à pas» a été réalisée afin de dégager les prédicteurs de l'ESA et du TSPT. Les potentiels prédicteurs utilisés dans le modèle sont les variables dont les résultats se sont avérés significatifs aux analyses statistiques univariées précédentes. Le seuil de signification a été fixé à 5%.

**Ethique**: tous les participants ont reçu des explications sur les objectifs et le déroulement de l'étude et ont accepté de participer au deux temps de l'étude en signant un consentement libre et éclairé.

## Résultats

Soixante-dix sujets ont été inclus dans notre série.

**Caractéristiques sociodémographiques et antécédents**: notre échantillon avait un âge moyen de 41,54 ans (écart-type =14,97). Le sex-ratio (H/F) était de 2,49 avec 50 sujets (71,4%) de sexe masculin. Quarante-un patient (58,6%) étaient mariés et 24 (34,3%) étaient célibataires. Quarante-huit patients (68,6%) résidaient en milieu urbain. Le niveau scolaire de notre série se répartissait comme suit: 6 sujets illettrés (soit 8,6%), 26 sujets (soit 37,1%) de niveau scolaire primaire, 35 (50%) de niveau scolaire secondaire, 3(4,3%) de niveau universitaire. Soixante-deux sujets (84,3%) étaient actifs sur le plan professionnel avant l'accident et 56 (80%) étaient d'un niveau socio-économique moyen. Vingt-neuf sujets (41,4%) avaient des antécédents médico-chirurgicaux. Quatre sujets avaient des antécédents psychiatriques personnels de dépression.

**Données relatives à l'accident**: cinquante-neuf accidentés (84,3%) étaient motorisés le jour de l'accident dont 44 étaient des conducteurs et 15 des non conducteurs. Onze sujets (15,7%) étaient des piétons. Nous avons réparti notre série en deux groupes de personnes: le groupe des actifs (les conducteurs) et le groupe des passifs (les non-conducteurs et les piétons). Vingt-sept personnes (38,6%) étaient accompagnées le jour de l'accident. Cinq sujets, soit 38,46% des conducteurs portaient leurs ceintures de sécurité. Tous les accompagnateurs ne portaient pas la ceinture de sécurité. Huit conducteurs (11,4%) roulaient avec une grande vitesse au moment de l'accident. Seize sujets (22,9%) avaient des lésions modérées ou sérieuses (score AIS <3) et 77,1% avaient des lésions sévères.

**Prévalence et facteurs de risque de l'ESA (Tableau 1 et Tableau 2):** vingt-six individus (37,1%) présentaient des symptômes de l'ESA. Les paramètres significativement associés à l'ESA ont été: le sexe féminin (p= 0,012), le niveau scolaire bas (p= 0,041), la présence d'antécédents personnels médico-chirurgicaux (p= 0,034), le fait d'être passif au décours de l'accident (p=0,026), la présence de lésions sévères selon l'échelle AIS (p= 0,02), et la présence d'une symptomatologie anxieuse (p= 0,01) et dépressive en T2 (p=0,035). Des scores faibles des stratégies de coping fonctionnelles (le coping actif (p<0,001), la planification (p<0,001), l'humour (p=0,047) et la réinterprétation positive (p=0,021)) ont été associés à la survenue de l'ESA. Des scores élevés des stratégies de coping dysfonctionnelles (la recherche de soutien social instrumental (p=0,006), la recherche de soutien social émotionnel (p=0,002), l'expression des sentiments (p<0,001), le blâme (p=0,002), le déni (p<0,001) et l'utilisation de substances (p=0,006)) ont été associés à la présence de l'ESA. L'analyse de régression logistique a montré que le niveau scolaire bas (p=0,024 ; OR=4,84), et la stratégie de coping de déni (p=0,013; OR=2,21) apparaissaient comme des facteurs de risque indépendants de l'ESA. La stratégie de coping de planification (p=0,01 ; OR=0,415) apparaissait comme un facteur protecteur.

**Tableau 1:** paramètres associés à la survenue de l’ESA

		ESA (+)	ESA(-)	P
**Âge**		45,73 (ET=14,53)	39,06 (ET=14,84)	0,079*
**Sexe**	**Masculin**	14(28 %)	36(72 %)	**0,012**
	**Féminin**	12(60 %)	8(40%)	
**Etat civil**	**Marié**	16(39 %)	25(61%)	0,698
	**Non marié**	10(34,5%)	19(65,5%)	
**Niveau scolaire**	**Analphabète/primaire**	16(50 %)	16(50 %)	**0,041**
	**Secondaire/supérieur**	10(26,3 %)	28(73,7%)	
**Milieu de résidence**	**Rural**	7(31,8%)	15(68,2%)	0,533
	**Urbain**	19(39,6 %)	29(60,4%)	
**Profession**	**Actif**	23(39%)	36(61%)	0,521
	**Inactif**	3(27,3%)	8(62,9%)	
**Niveau socioéconomique**	**Bas**	6(42,9 %)	8(57,1%)	0,621
	**Moyen**	20(35,7 %)	36(64,3%)	
**Antécédents Médicochirurgicaux**	**Oui**	15(51,7 %)	14(48,3 %)	**0,034**
	**Non**	11(26,8%)	30(73,2%)	
**Antécédents psychiatriques**	**Oui**	4(44,4%)	5(55,6%)	0,718
	**Non**	22(36,1%)	39(63,9%)	
**Responsabilité dans l´ accident**	**Actif**	12(46,2 %)	32(72,7 %)	**0,026**
	**Passif**	14(53,8 %)	12 (46,2%)	
**Sujets Accompagnants**	**Oui**	12(44,4%)	15(55,6%)	0,316
	**Non**	14(32,6 %)	29(67,4%)	
**Port de ceinture de sécurité**	**Oui**	3(11,5%)	23(88,5 %)	0,272
	**Non**	2(4,5%)	42(95,5 %)	
**Grande vitesse**	**Oui**	2(25 %)	6(75 %)	0,701
	**Non**	24(38,7 %)	38(61,3%)	
**Score AIS**	**<3**	2(12,5 %)	14(87,5 %)	**0 ,020**
	**≥ 3**	24(44,4 %)	30(55,6 %)	
**Anxiété**	**Oui**	18(58,1 %)	13(41,9%)	**0,001**
	**Non**	8(20,5%)	31(79,5%)	
**Dépression**	**Oui**	18(48,6%)	25(75,8%)	**0,035**
	**Non**	8(24,2%)	19(51,4%)	

* Test de Mann-Whitney

**Tableau 2:** lien entre les stratégies de coping et la survenue de l'ESA et du TSPT

	ESA			TSPT		
	**Oui**	**Non**	**p**	**Oui**	**Non**	**p**
**Coping actif**	2,46	4,34	<0,001*	2,32	4,52	<0,001*
**Planification**	2,96	4,27	<0,001*	2,85	4,4	<0,001*
**Recherche de soutien social instrumental**	4,07	3,29	0,006*	4,25	3,14	<0,001*
**Recherche de soutien social émotionnel**	5,11	3,61	0,002*	5,53	3,26	<0,001*
**Expression des sentiments**	5,26	2,95	<0,001*	5,5	2,69	<0,001*
**Désengagement comportemental**	3,19	2,97	0,744*	3	3,09	0,445*
**Distraction**	3,57	3,13	0,091*	3,6	3,09	0,049*
**Blâme**	3,5	2,84	0,002*	3,78	2,61	<0,001*
**Réinterprétation positive**	2,3	2,86	0,021*	2,14	3	<0,001*
**Humour**	3,23	3,43	0,047*	3,21	3,45	0,027*
**Déni**	3,5	2,52	<0,001*	3,75	2,3	<0,001*
**Acceptation**	2,96	3,86	0,351*	2,75	4,04	0,04*
**Religion**	4,53	5,02	0,05*	4,71	4,92	0,356*
**Utilisation de substances**	3,84	3,13	0,006*	3,92	3,04	0,001*

* Test de Mann-Whitney

**Prévalence et facteurs de risque du TSPT (Tableau 2 et Tableau 3):** vingt-huit patients (40%) présentaient des symptômes du TSPT. Les paramètres associés à la survenue d'un TSPT ont été : la résidence en milieu urbain (p=0,012), le fait d'être passif au décours de l'accident (p=0,02), et la présence d'une symptomatologie anxieuse (p=0,001) et dépressive (p=0,002). Des scores faibles des stratégies de coping fonctionnelles (le coping actif (p<0,001), la planification (p<0,001), l'humour (p=0,027), la réinterprétation positive (p<0,001), et l'acceptation (p=0,004)) ont été associés à la survenue du TSPT. Des scores élevés des stratégies de coping dysfonctionnelles (la recherche de soutien social émotionnel (p<0,001), la recherche de soutien social instrumental (p<0,001), l'expression des sentiments (p<0,001), la distraction (p=0,049), le blâme (p<0,001), le déni (p<0,001), l'utilisation de substances (p=0,001)) ont été associés à la survenue du TSPT. Après analyse de régression logistique, la résidence en milieu urbain (p=0,004 ; OR=8,26) et la présence d'une symptomatologie anxieuse (p=0,037, OR= 3,63) et dépressive (p=0,01, OR= 5,51) apparaissent comme des facteurs de risque indépendants du TSPT. Concernant le lien entre l'ESA et le TSPT, 53,6% du groupe présentant un ESA en T1 avaient en T2 un TSPT; versus 46,4% qui n'avaient pas un TSPT. Cette différence a été statistiquement significative (p=0,002).

**Tableau 3:** paramètres associés à la survenue du TSPT

		TSPT (+)	Non TSPT (-)	P
**Age**		43,39 (ET=15,87)	40,3 (ET=14,4)	0,403*
**Sexe**	Masculin	19 (38 %)	31 (62 %)	0,589
	Féminin	9(45 %)	11(55 %)	
**Etat civil**	Marié	17(41,5 %)	24(58,5 %)	0,766
	Non marié	11(37,9 %)	18(62,1 %)	
**Niveau scolaire**	Analphabète/primaire	10 (31,2 %)	22 (68,8 %)	0,17
	Secondaire/supérieur	18(47,4 %)	20(52,6 %)	
**Milieu de résidence**	Rural	4(18,2 %)	18(81,8 %)	0,012
	Urbain	24(50 %)	24(50 %)	
**Profession**	Actif	26(44,1 %)	33(55,9 %)	0,18
	Inactif	2 (18,2 %)	9 (81,8 %)	
**Niveau Socioéconomique**	Bas	5 (37,5 %)	9(64,3 %)	0,714
	Moyen	23(41,1%)	33(58,9 %)	
**Antécédents Médicochirurgicaux**	Oui	15(51,7 %)	14(48,3 %)	0,092
	Non	13(31,7 %)	28 (68,3 %)	
**Antécédents psychiatriques**	Oui	1 (11,1 %)	8(88,9 %)	0,075
	Non	27(44,3 %)	34(55,7%)	
**Notion de Responsabilité**	Actif	13(46,4 %)	31(73,8 %)	0,020
	Passif	15(53,6 %)	11(26,2 %)	
**Sujets Accompagnants**	Oui	12 (44,4 %)	15 (55,6%)	0,316
	Non	14(32,6 %)	29 (67,4%)	
**Port de ceinture de sécurité**	Oui	1(3,6 %)	27(96,4%)	0,343
	Non	4(9,5%)	38(90,5 %)	
**Grande Vitesse**	Oui	2(25 %)	6(75 %)	0,462
	Non	26 (41,9 %)	36 (58,1 %)	
**Score AIS**	<3	5(31,2 %)	11(68,8 %)	0 ,416
	≥ 3	23(42,6 %)	31(57,4%)	
**Anxiété**	Oui	19(61,3 %)	12(38,7%)	0,001
	Non	9(23,1 %)	30(76,9 %)	
**Dépression**	Oui	21 (56,8%)	16 (43,2 %)	0,002
	Non	7 (21,2%)	26 (78,8 %)	
**TSA**	Oui	15(53,6%)	13(46,4 %)	0,002
	Non	11(26,2%)	31(73,8%)	

* Test de Mann-Whitney

## Discussion

Notre étude s'est intéressée à l'étude de la prévalence de l'ESA et du TSPT et de leurs facteurs prédicteurs dans une population de victimes d'AC. Cet intérêt a été suscité par la fréquence des AC en Tunisie et leur influence négative sur la santé physique et psychique avec une détérioration des conditions de vie et des problèmes sociaux et économiques. Néanmoins, les symptômes de l'ESA et du TSPT ont été relevé à l'aide d'échelles psychométriques. Des entretiens semi-structurés auraient été nécessaires pour retenir ces diagnostics. Une revue des données de la littérature a montré que la moyenne des prévalences de l'ESA chez les survivants d'AC estimée par des auto-questionnaires (17,82%) a été significativement plus élevée que ceux estimées par des entretiens semi- structurés (15,26%) [11]. Pourtant, certaines études ont montré que les auto-questionnaires comme le PTSD-checklist ont une bonne spécificité et sensibilité [12,13]. La durée de 6 semaines après l'accident est courte pour étudier l'évolution des symptômes psycho-traumatiques.

Dans notre étude, les prévalences de l'ESA et du TSPT; respectivement 37,1% et 40% ; étaient supérieures à la plupart des prévalences trouvées dans la littérature [14,15]. La prévalence de l'ESA variait dans la littérature entre 9% [16] et 42% [17], et celle du TSPT variait entre 15,4% [18] et 46,5% [15]. Nos résultats plus élevés que ceux retrouvés dans la littérature peuvent être expliqués par la nature hospitalière de notre série et par les disparités génétiques, culturelles et sociales entre les différents pays [15,17]. D'une part, l'hospitalisation après le traumatisme, accroit le stress ressenti par les patients [14]. D'autre part, le profil génétique des individus explique environ 30% des manifestations symptomatiques du TSPT dans une étude effectuée sur des jumeaux [16].

La survenue d'un ESA était plus fréquente chez les sujets de sexe féminin (p=0,021) dans notre série. La majorité des études affirmaient que les femmes auront un risque plus important de développer des troubles psycho-traumatiques [11,16,19,20]. Cette vulnérabilité pourrait être expliquée par les différences entre les deux sexes. En fait, les traumatismes de l'enfance sont plus fréquents chez les femmes [21] avec une proportion élevée des femmes non conductrices [22]. Les femmes ont des perceptions de menace et de perte de contrôle plus importantes ; et une forte prévalence de la dépression à la suite des accidents de la route et qui est associée à un risque élevé d'ESA [22]. Les hommes n'exprimaient pas facilement leur souffrance psychologique. Certains facteurs neurobiologiques peuvent expliquer cette différence liée au genre. L'effet réducteur de stress de l'ocytocine serait plus prédominant chez les femmes ayant subi un traumatisme psychologique, et son effet serait accru par l'œstrogène [23]. Cependant, une déficience du signal glucocorticoïde observée dans le TSPT et aggravée par l'ocytocine chez la femme entrainait une persistance de la réaction de stress [24]. De plus, un niveau scolaire bas constituait un facteur de risque indépendant de la survenue d'un ESA (p=0,024 ; OR=4,84). La plupart des études considéraient qu'un niveau d'éducation faible soit un facteur de risque pour des troubles psycho-traumatiques [25-27]. Il serait associé à des distorsions cognitives et à des stratégies de coping dysfonctionnelles. Le déficit cognitif limite les capacités de résolution de problèmes avec un recours à des stratégies de coping centré sur les émotions [26]. De plus, Verger et coll. affirmaient que les sujets ayant un niveau scolaire supérieur se distinguent par une plus grande sérénité [28].

Être résident en zone urbaine constituait également un facteur de risque indépendant du TSPT (p=0,004 ; OR=8,26). Ceci pourrait être expliqué par le niveau élevé de stress perçu chez ces sujets, et à une moindre capacité d'adaptation face aux traumatismes psychiques [29]. Les patients ayant des antécédents personnels médico-chirurgicaux étaient plus vulnérables à développer un ESA (p=0,034). La présence d'antécédents somatiques a été associée à la survenue d'un TSPT, et pourrait constituer un facteur de moins bon pronostic [30]. En fait, cette comorbidité somatique pourrait péjorer l'évolution de la pathologie psychiatrique en masquant certains de ses symptômes et en rendant le diagnostic plus difficile et de ce fait retarder la prise en charge et constituer un facteur de résistance thérapeutique [30]. Nous avons noté également une association statistiquement significative entre le fait d'être passif (non conducteur ou piéton) au cours de l'accident et la survenue de l'ESA et du TSPT (p respectifs de 0,026 et 0,02). Dans ce sens, certains auteurs affirmaient qu'occupant la position de non conducteur soit un facteur prédictif de survenue de troubles post traumatiques [31-33]. Ceci pourrait être expliqué par le fait que la personne se sent généralement victime, et qu'elle a le sentiment de perte de contrôle sur ce qui lui arrive.

Concernant la sévérité des lésions, la gravité des lésions évaluée par l'AIS a été significativement liée à la survenue de l'ESA (p=0,02). Winston *et al*. ont montré que la gravité des lésions a été un facteur prédictif indépendant de survenue de l'ESA chez les enfants victimes d'accidents de la circulation (OR= 6,95) [34]. Plusieurs études ont mis en évidence un lien entre la sévérité des lésions et le TSPT [16, 19,31-33]. Ceci a été expliqué par le fait que la sévérité des lésions a été associée à des réactions de stress initial plus importantes, ce qui est prédictif de la survenue plus rapide et durable d'un TSPT. Paradoxalement, les conséquences psychologiques des traumatismes dus aux AC ne sont pas toujours proportionnelles à la gravité des blessures : même les traumatismes relativement mineurs pourraient avoir des répercussions psychosociales profondes. C'est le vécu cognitif et émotionnel de la situation qui pourrait prédire la survenue d'un TSPT [35]. Ceci serait dû au fait que l'évaluation objective de la sévérité des lésions n'est pas nécessairement un indicateur de la perception du sujet de la mise en jeu de son pronostic vital [35]. Une symptomatologie anxieuse a été liée à la survenue d'un ESA et d'un TSPT dans notre étude et constituait un facteur de risque indépendant du TSPT (p=0,037, OR= 3,63). Plusieurs études ont montré que certains troubles anxieux peuvent être comorbides aux troubles liés aux traumatismes [29,34,36]. Ils peuvent revêtir diverses formes: anxiété généralisée, crises d'angoisse aiguës, trouble panique ou encore troubles phobiques [2,37]. Il a été également démontré que les individus ayant une prédisposition aux troubles anxieux sont plus vulnérables à développer des réponses cliniquement significatives aux événements de vie stressants [34,36]. On peut assister à une progressive différenciation de la symptomatologie du TSPT et à son remplacement par des troubles anxieux dont le lien avec l'évènement traumatique n'est plus immédiatement perceptible. La prévalence des troubles anxieux chroniques (estimée de 15 à 20%) constatés plusieurs années après un événement traumatique, témoignait de ce processus [28].

De plus, la symptomatologie dépressive a été associée à la survenue de l'ESA et du TSPT et constituait un facteur de risque indépendant du TSPT (p=0,01, OR= 5,51). Certains auteurs ont rapporté que la dépression a été associée à l'ESA mais ne constituait pas un élément prédicteur de sévérité [29,38]. Elle serait la conséquence de l'exposition à l'événement traumatique et ne constitue pas un facteur de vulnérabilité [39]. Elle apparait également comme le trouble le plus associé au TSPT bien qu'ils soient deux pathologies différentes [40] avec une prévalence de cette comorbidité de 20 à 61% [18,36,39]. Quand elle est constatée dans les suites immédiates du traumatisme, elle augmente le risque de survenue de TSPT avec passage à la chronicité. Elle peut être lié aux séquelles et aux pertes induites par l'accident (intégrité physique, autonomie, travail, perte d'un proche …) ou à la présence d'un ESA non ou insuffisamment traitée [40,41]. Concernant les stratégies de coping fonctionnelles, un score faible du coping actif ; définie par la capacité à se retenir d'agir prématurément et à attendre le moment idéal pour passer à l'action ; a été associé à la survenue d'un ESA et du TSPT dans notre série. Cette stratégie peut aider les individus à gérer le traumatisme et le stress et à prévenir les troubles émotionnels et psychologiques et surtout le TSPT [42]. La stratégie de planification ; qui consiste à réfléchir à la façon de gérer le stresseur et aux étapes pour y arriver ; apparait comme un facteur protecteur de survenue de l'ESA. Elle serait associée à un plus faible niveau de stress perçu et de détresse psychologique [8]. Dans notre étude ainsi que dans la littérature, le recours à la stratégie à l'humour était plus fréquent chez les sujets ne présentant pas un TSPT. Elle permet de diminuer la perception de menace et peut générer une mise à distance par la pensée grâce à une exagération des aspects positifs, de l'humour ou une sous-estimation de la situation. Cette stratégie est largement utilisée par les professionnels durant leur mission de secours vu que c'est le seul moyen de mettre en récit la souffrance de façon à ce qu'elle soit socialement acceptable [3]. Pour les stratégies de coping dysfonctionnelles, un score élevé de la stratégie d'expression des sentiments (se concentrer et à exprimer la détresse causée par le stresseur) a été lié à la survenue d'un ESA et du TSPT dans notre série. Cette réaction émotionnelle que tend à éviter et à éliminer les cognitions négatives et les mémoires du traumatisme entraine une activation de la mémoire émotionnelle et stimule l'anxiété [43,44]. Le recours à la stratégie de déni ou d'évitement cognitif (refuser de croire que le stresseur existe ou à agir comme s'il n'existait pas) apparait comme un facteur de risque indépendant de survenue du TSPT. Plusieurs études ont montré que l'évitement après l'événement traumatique constitue un des facteurs prédicteurs de survenue et de gravité de l'ESA et du TSPT chez les survivants d'accidents de la circulation [31,38,43]. En effet, l'évitement est associé à un mauvais comportement de recherche d'aide. La minimisation du comportement d'évitement après un AC peut faciliter l'ajustement aux stimuli redoutés, et ainsi entraîner une réduction des symptômes intrusifs [45]. La résolution de l'expérience traumatique est conditionnée par l'acceptation des émotions aversives, et l'habituation au stress.

Dans notre série, l'ESA constitue un facteur prédisposant à la survenue d'un TSPT. Il a été identifié dans plusieurs études comme le facteur le plus prédicteur de développement du TSPT [17,38,46]. Cette valeur prédictive a été expliquée par certaines théories neurobiologiques et cognitivo-comportementale [22,47]. L'activation intense du système nerveux sympathique suite à l'exposition à un évènement traumatique et la libération de taux très élevés de glucocorticoïdes auront des conséquences délétères à long terme au sein de régions cérébrales impliquées dans les capacités mnésiques (hippocampe) et de régulation émotionnelle (amygdale et cortex préfrontal). En parallèle, l'activité du cortex pré-préfrontal se réduit bloquant toute possibilité d'analyse de la situation et de régulation des régions sous-corticales [22,47]. Le maintien de l'organisme en état de stress prolongé modifie de manière durable l'activité des structures cérébrales régulatrices de la réaction de stress au niveau cognitif, émotionnel et biologique et entraine le passage à la chronicité avec installation d'un TSPT. Pour la théorie cognitivo-comportementale, l'ESA serait d'abord provoqué par un conditionnement classique de la peur. Des indices (objets, lieux sensations et odeurs) vont acquérir les propriétés aversives de l'évènement traumatique et provoquer une réponse anxieuse similaire à celle provoquée par celui-ci. Parallèlement, un conditionnement opérant pousse l'individu à tout faire pour éviter d'être confronté à ces stimuli (fuir une situation ou un lieu, éviter le travail, etc.). Par la suite, le traitement de l'information de l'évènement traumatique s'organise en un réseau de la peur ; l'association et le pouvoir d'excitation des stimuli associés à la peur sont renforcés. Les processus de mémorisation et d'association qui traitent l'information vont créer un biais attentionnel vers les stimuli associés à l'évènement traumatique et la perception de menace sera toujours activée et se généralisera ; ce qui expliquerait les symptômes d'évitement et les pensées intrusives du TSPT [22,47].

## Conclusion

Notre étude a montré que les prévalences de l'ESA et du TSPT chez les accidentés de la voie publique sont élevées. L'analyse de régression logistique a montré que le niveau scolaire bas, et la stratégie de coping de déni apparaissaient comme des facteurs de risque indépendants de l'ESA. La résidence en milieu urbain, la présence d'un niveau élevé d'anxiété et de dépression apparaissaient comme des facteurs de risque indépendants du TSPT. Ces éléments nous ont permis de déterminer un profil de personnes plus exposées que d'autres à ces troubles, facilitant ainsi le dépistage des sujets à risque par le personnel soignant. L'ensemble des personnels de soins devrait être capable de repérer et d'orienter les patients présentant ou susceptibles de présenter ces troubles afin de mettre en place des traitements dont l'efficacité a été prouvée et donc d'éviter les effets néfastes de la maladie qui concernent non seulement le patient, mais aussi son entourage. Ce dépistage permettrait dans un premier temps, d'informer les patients de la possibilité de survenue de tels troubles et, dans un deuxième temps, d'améliorer la prise en charge médicale et sociale.

### Etat des connaissances sur le sujet

- *Les accidents de la circulation sont fréquents en Tunisie et d'après les chiffres communiqués par l'Observatoire National de la Sécurité Routière (ONSR), 4726 accidents de la circulation ont eu lieu durant l'année 2020, ayant causé 938 décès et 6719 blessés;*
- *Les accidents de circulation par leur impact physique (subir une blessure grave ou être menacé de mort, être témoin des blessures graves, de la mort, ou de la menace de mort infligée à d'autres personnes) ont des conséquences psychologiques fréquentes telles que le trouble stress aigu et le trouble stress post-traumatique;*
- *Plusieurs facteurs favorisant la survenue de troubles post-traumatiques ont été identifiés*.

### Contribution de notre étude à la connaissance

- *La prévalence de l'ESA chez les victimes d'accidents de circulation et son pouvoir prédicteur du TSPT ont été peu abordés dans la littérature chez les patients victimes d'accidents de la circulation*.
- *L'étude des stratégies de coping face à un traumatisme psychique constitue l'un des points forts de notre étude;*
- *La régression logistique a mis en évidence certains facteurs de risque indépendants de survenue de l'ESA et du TSPT*.

## Remerciements

Nous remercions tous les patients qui ont accepté de participer à l'étude ainsi que le personnel médical et paramédical du service de chirurgie orthopédique et traumatologie du Centre Hospitalo-Universitaire de Sfax, Tunisie.

## References

[1] World Health Organisation (2020). Accidents de la route: principaux repères.

[2] O'Donnell ML, Creamer M, Pattison P, Atkin C (2004). Psychiatric morbidity following injury. Am J Psychiatry.

[3] Bryant RA (2018). The Current Evidence for Acute Stress Disorder. Curr Psychiatry Rep.

[4] American Psychiatric Association (2013). Diagnostic and Statistical Manual of Mental Disorders.

[5] Traber D (2019). Comment prévenir le trouble de stress post traumatique?. Reflexion et application chez les professionnels à risques de la région Auvergne Rhône-Alpes.

[6] Van Ditshuizen JC, Sewalt CA, Palmer CS, Van Lieshout EMM, Verhofstad MHJ, Den Hartog D (2021). The definition of major trauma using different revisions of the abbreviated injury scale. Scand J Trauma Resusc Emerg Med.

[7] Bryant RA, Moulds ML, Guthrie RM (2000). Acute Stress Disorder Scale: a self-report measure of acute stress disorder. Psychol Assess.

[8] Muller L, Spitz E (2003). Évaluation multidimensionnelle du coping : validation du Brief COPE sur une population française. Encéphale.

[9] Terkawi A, Tsang S, AlKahtani G, Al-Mousa S, Al Musaed S, AlZoraigi U (2017). Development and validation of Arabic version of the Hospital Anxiety and Depression Scale. Saudi J Anaesth.

[10] Zigmond AS, Snaith RP (1983). The hospital anxiety and depression scale. Acta Psychiatr Scand.

[11] Dai W, Liu A, Kaminga AC, Deng J, Lai Z, Yang J (2018). Prevalence of acute stress disorder among road traffic accident survivors: a meta-analysis. BMC Psychiatry.

[12] Coffey SF, Gudmundsdottir B, Beck JG, Palyo SA, Miller L (2006). Screening for PTSD in motor vehicle accident survivors using the PSS-SR and IES. J Trauma Stress.

[13] Karstoft KI, Andersen SB, Bertelsen M, Madsen T (2014). Diagnostic accuracy of the posttraumatic stress disorder checklist-civilian version in a representative military sample. Psychol Assess.

[14] Yaşan A, Guzel A, Tamam Y, Ozkan M (2009). Predictive factors for acute stress disorder and posttraumatic stress disorder after motor vehicle accidents. Psychopathology.

[15] Van den Heuvel L, Suliman S, Malan-Müller S, Hemmings S, Seedat S (2016). Brain-derived neurotrophic factor Val66met polymorphism and plasma levels in road traffic accident survivors. Anxiety Stress Coping.

[16] Fekadu W, Mekonen T, Belete H, Belete A, Yohannes K (2019). Incidence of post-traumatic stress disorder after road traffic accident. Front Psychiatry.

[17] Hamanaka S, Asukai N, Kamijo Y, Hatta K, Kishimoto J, Miyaoka H (2006). Acute stress disorder and posttraumatic stress disorder symptoms among patients severely injured in motor vehicle accidents in Japan. Gen Hosp Psychiatry.

[18] Bedaso A, Kediro G, Ebrahim J, Tadesse F, Mekonnen S, Gobena N (2020). Prevalence and determinants of post-traumatic stress disorder among road traffic accident survivors: a prospective survey at selected hospitals in southern Ethiopia. BMC EmJuinMed.

[19] Kovacevic J, Miskulin M, Degmecic D, Vcev A, Leovic D, Sisljagic V (2020). Predictors of mental health outcomes in road traffic accident survivors. J Clin Med.

[20] Yohannes K, Gebeyehu A, Adera T, Ayano G, Fekadu W (2018). Prevalence and correlates of post-traumatic stress disorder among survivors of road traffic accidents in Ethiopia. Int J Ment Health Syst.

[21] Anda RF, Felitti VJ, Bremner JD, Walker JD, Whitfield C, Perry BD (2006). The enduring effects of abuse and related adverse experiences in childhood. Eur Arch Psychiatry Clin Neurosci.

[22] Bryant RA, Harvey AG (2003). Gender differences in the relationship between acute stress disorder and posttraumatic stress disorder following motor vehicle accidents. Aust N Z J Psychiatry.

[23] Taylor SE, Klein LC, Lewis BP, Gruenewald TL, Gurung RA, Updegraff JA (2000). Biobehavioral responses to stress in females: tend-and-befriend, not fight-or-flight. Psychol Rev.

[24] Yehuda R (2009). Status of Glucocorticoid Alterations in Post-traumatic Stress Disorder. Ann N Y Acad Sci.

[25] Dougall AL, Ursano RJ, Posluszny DM, Fullerton CS, Baum A (2001). Predictors of posttraumatic stress among victims of motor vehicle accidents. Psychosom Med.

[26] Suliman S, Stein DJ, Seedat S (2014). Clinical and Neuropsychological Predictors of Posttraumatic Stress Disorder. Medicine (Baltimore).

[27] Stein DJ, Karam EG, Shahly V, Hill ED, King A, Petukhova M (2016). Post-traumatic stress disorder associated with life-threatening motor vehicle collisions in the WHO World Mental Health Surveys. BMC Psychiatry.

[28] Verger P, Bard D, Noiville C, Lahidji R, French Committee for Prevention and Precaution (2008). Environmental disasters: preparing for impact assessments and operational feedback. Am J Disaster Med.

[29] Suliman S, Troeman Z, Stein DJ, Seedat S (2013). Predictors of acute stress disorder severity. J Affect Disord.

[30] Ongecha-Owuor FA, Kathuku DM, Othieno CJ, Ndetei DM (2004). Post traumatic stress disorder among motor vehicle accident survivors attending the orthopaedic and trauma clinic at Kenyatta National Hospital, Nairobi. East Afr Med J.

[31] Chossegros L, Hours M, Charnay P, Bernard M, Fort E, Boisson D (2011). Predictive factors of chronic post-traumatic stress disorder 6 months after a road traffic accident. Accid Anal Prev.

[32] Tournier C, Charnay P, Tardy H, Chossegros L, Carnis L, Hours M (2014). A few seconds to have an accident, a long time to recover: consequences for road accident victims from the ESPARR cohort 2 years after the accident. Accid Anal Prev.

[33] Matsuoka Y, Nishi D, Nakajima S, Kim Y, Homma M, Otomo Y (2008). Incidence and prediction of psychiatric morbidity after a motor vehicle accident in Japan: the Tachikawa Cohort of Motor Vehicle Accident Study. Crit Care Med.

[34] Winston FK, Baxt C, Kassam-Adams NL, Elliott MR, Kallan MJ (2005). Acute traumatic stress symptoms in child occupants and their parent drivers after crash involvement. Arch Pediatr Adolesc Med.

[35] Heron-Delaney M, Kenardy J, Charlton E, Matsuoka Y (2013). A systematic review of predictors of posttraumatic stress disorder (PTSD) for adult road traffic crash survivors. Injury.

[36] Kessler RC, Sonnega A, Bromet E, Hughes M, Nelson CB (1995). Posttraumatic stress disorder in the National Comorbidity Survey. Arch Gen Psychiatry.

[37] Mayou R, Bryant B, Duthie R (1993). Psychiatric consequences of road traffic accidents. BMJ.

[38] Bryant RA, Harvey AG (1998). Relationship between acute stress disorder and posttraumatic stress disorder following mild traumatic brain injury. Am J Psychiatry.

[39] Breslau N, Davis GC, Andreski P, Peterson E (1991). Traumatic events and posttraumatic stress disorder in an urban population of young adults. Arch Gen Psychiatry.

[40] Shalev AY, Freedman S, Peri T, Brandes D, Sahar T, Orr SP (1998). Prospective study of posttraumatic stress disorder and depression following trauma. Am J Psychiatry.

[41] Pailler ME, Kassam-Adams N, Datner EM, Fein JA (2007). Depression, acute stress and behavioral risk factors in violently injured adolescents. Gen Hosp Psychiatry.

[42] Olff M, Langeland W, Draijer N, Gersons BPR (2007). Gender differences in posttraumatic stress disorder. Psychol Bull.

[43] Thompson NJ, Fiorillo D, Rothbaum BO, Ressler KJ, Michopoulos V (2018). Coping strategies as mediators in relation to resilience and posttraumatic stress disorder. J Affect Disord.

[44] Kucmin T, Kucmin A, Turska D, Turski A, Nogalski A (2018). Coping styles and dispositional optimism as predictors of post-traumatic stress disorder (PTSD) symptoms intensity in paramedics. Psychiatr Pol.

[45] Bryant RA, Marosszeky JE, Crooks J, Baguley I, Gurka J (2000). Coping style and post-traumatic stress disorder following severe traumatic brain injury. Brain Inj.

[46] Murray J, Ehlers A, Mayou RA (2002). Dissociation and post-traumatic stress disorder: two prospective studies of road traffic accident survivors. Br J Psychiatry J Ment Sci.

[47] Bryant RA, Creamer M, O'Donnell M, Silove D, McFarlane AC (2012). The capacity of acute stress disorder to predict posttraumatic psychiatric disorders. J Psychiatr Res.

